# The Mechanism Exploration of Follicular Fluids on Granulose Cell Apoptosis in Endometriosis-Associated Infertility

**DOI:** 10.1155/2021/6464686

**Published:** 2021-10-28

**Authors:** Lu Chen, Zhexin Ni, Zailong Cai, Wen Cheng, Shuai Sun, Chaoqin Yu, JinYu

**Affiliations:** ^1^Department of Gynecology of Traditional Chinese Medicine, Changhai Hospital, PLA Naval Medical University, Shanghai 200433, China; ^2^Department of Traditional Chinese and Western Medicine, Zhejiang Chinese Medicine Association, Zhejiang, 31000, China; ^3^Department of Biochemistry and Molecular Biology, PLA Naval Medical University, Shanghai 200433, China; ^4^International Peace Maternity and Child Health Hospital, School of Medicine, Shanghai Jiao Tong University, Shanghai 200025, China

## Abstract

**Objective:**

To explore the mechanisms of follicular fluids (FFs) on granulose cell (GC) apoptosis in endometriosis-associated infertility.

**Materials and Methods:**

60 infertile women were enrolled. The FFs from 30 endometriosis-associated infertility (EI) patients were collected and processed by ELISA hormone assay and proteomic profiling. The ovary GCs collected from 30 tubal-associated infertility (TI) patients were cultured in follicular fluids of endometriosis-associated infertility patients (EI-FFs), and the apoptosis mechanisms were explored by flow cytometry assay, real-time PCR, Western blotting, and protein–protein interaction (PPI) network analysis.

**Results:**

Our results showed that the expression of 22 specific proteins was significantly different in the FFs from EI and TI patients, and the level of testosterone and anti-Müllerian hormone was not obviously different between the two groups. EI-FFs could accelerate the apoptosis process of granulose cells of tubal-associated infertility patients (TI-GCs) by regulating the expression of 5 apoptosis-related proteins including BCL2, BAX, CASP3, CASP9, and TP53. The correlation of these 22 specific proteins and 5 apoptosis-related proteins was analyzed by PPI, and 5 protein biomarkers (INS, CXCL10, ICAM1, WIF1, and TNFRSF13C) and 5 signaling pathways (cytokine-cytokine receptor interaction, apoptosis, regulation of actin cytoskeleton, MAPK, and p53 signaling pathway) were predicted.

**Conclusion:**

This research clarified the effect and explored the mechanisms of EI-FFs on the apoptosis of TI-GCs and indicated the protein biomarkers and signaling pathways for further study.

## 1. Introduction

Endometriosis is a common dynamic complex estrogen-dependent gynecological disorder characterized by the presence of functional endometrial-type mucosa outside the uterine cavity that affects up to 50% of infertile women of reproductive age [1]. At present, although Assisted Reproductive Technology (ART) is the main treatment for endometriosis-associated infertility, the number of eggs obtained and the rate of fertilization and pregnancy are lower compared with the nonendometriosis patients. In addition to pelvic structure changes and immune deficiency, the altered follicular microenvironment in endometriosis patients is one of the most important contributing factors to infertility [2].

Granulosa cells (GCs), which surround the oocytes to form the follicular structure, regulate the occurrence, growth, maturation, and atresia of the follicle. The morphological and functional changes of GCs are important markers of follicular development. Recently, researchers have gradually realized that the basic pathological mechanism of follicular dysplasia or atresia is apoptosis [3], and the apoptosis of GCs is regulated by various cytokines, hormones, and other factors, involving the changes of multiple signaling pathways. Follicular fluids (FFs), closely linked to the growth of GCs, are the critical microenvironment for follicular maturation. However, the components of FFs and the exact mechanisms are still unclear. Recent studies have shown that FFs in endometriosis-associated infertility patients (EI-FFs) have qualitative and quantitative changes in the pathophysiological process of the disease [4], which may be an important role for inhibiting GC proliferation and inducing GC apoptosis, thus leading to infertility in endometriosis patients. Therefore, studying the effects and possible mechanisms of EI-FFs on GC apoptosis may help develop new strategies to improve follicle quality and pregnancy outcome.

## 2. Material and Methods

### 2.1. Study Approval

This study was approved and monitored by the ethics committee of the Changhai Hospital, PLA Naval Medical University. Informed written consent was obtained from each patient before sample collection.

### 2.2. Patient Selection

We enrolled a total of 60 infertile women, including 30 patients with both laparoscopic and histological diagnoses of deeply infiltrating endometriosis (stages I-II according to the American Society for Reproductive Medicine 1997) and 30 controls with tubal-associated infertility (TI) at the Assisted Reproductive Centre of the Changhai Hospital, PLA Naval Medical University.

#### 2.2.1. Inclusion Criteria

The inclusion criteria were as follows: (1) age < 35 years; (2) a history of infertility defined as failure to establish a clinical pregnancy after 1 year of regular, unprotected sexual intercourse [5]; (3) both basal serum follicle-stimulating hormone (FSH) and luteinizing hormone (LH) levels measured on day 3 of the menstrual cycle were 4~10 IU/L before controlled ovarian hyperstimulation; and (4) patients have not received any hormones within 6 months.

#### 2.2.2. Exclusion Criteria

Exclusion criteria were as follows: (1) infertility caused by chromosomal abnormalities; (2) polycystic ovary syndrome or pure high androgen levels, diabetes, thyroid diseases, and any other untreated or insufficiently corrected endocrinopathies; (3) premature ovarian failure, ovarian tumor, uterus diseases, or any previous history of tuberculosis, pelvic surgery, radiotherapy, and chemotherapy that may affect ovarian function; (4) liver, kidney, heart, or blood diseases; and (5) smoking, alcoholism, or drug addiction, etc.

### 2.3. Controlled Ovarian Stimulation

To reduce the impacts of potential confounding factors between groups, all patients were treated with identical protocols for controlled ovarian stimulation with standard gonadotrophin-releasing hormone agonist combined with human menopausal gonadotropin (hMG) protocol [6]. A single injection of human chorionic gonadotropin (hCG) was given when at least three follicles reached a minimum diameter of 18-20 mm. Oocytes were retrieved 36 hours later using a standard ultrasonically guided follicular puncture.

### 2.4. Sample Collection and Processing

FFs of each follicle in all patients were aspirated separately into each tube. FFs from follicles smaller than 15 mm, follicles without an egg, follicles with more than 1 oocyte, and FFs with blood contamination were discarded. The FF samples were centrifuged (2000 g for 20 min at 4°C), and the supernatant was frozen at -80°C [7]. Ovary GCs from tubal-associated infertility patients (TI-GCs) were washed twice in ice-cold PBS and centrifuged (1000 g for 15 min at 4°C). The cell precipitation was suspended in the culture medium, layered over a 50% Percoll; the PBS solution then absorbed the white cloud-like boundary layer after centrifugation and collected the GCs in the boundary layer. Erythrocyte lysate was centrifuged for 10 minutes at 2000 g to remove red blood cells and wash them with PBS. The collected TI-GCs were maintained at 37°C in a humidified incubator containing 5% CO_2_ in DMEM/nutrient mixture F-12 Ham (HyClone, USA) supplemented with 10% fetal bovine serum (HyClone, USA), 100 IU/mL of penicillin (Gibco, USA), and 0.1 mg/mL of streptomycin (Gibco, USA).

### 2.5. ELISA Hormone Assay

The hormone levels of anti-Müllerian hormone (AMH) and testosterone (T) of each FF in all patients were analyzed by ELISA. Each analysis was conducted according to the manufacturer's protocol (Antibodies-online; AMH product number ML-Elisa-0181, T product number SBJ-H002).

### 2.6. Proteomic Profiling

Protein microarray was purchased from RayBiotech, USA (product number, AAH-BLG-3507). According to the instructions, the FF samples were diluted with 1∗PBS (pH = 8.0) and dialyzed with 500 mL PBS; the protein array membranes were blocked in a blocking buffer for 30 min and then incubated with samples at 4°C overnight. After being incubated with diluted biotin-conjugated antibodies for 1-2 h at room temperature, the membranes were reacted with streptavidin-conjugated Fluor and scanned with an Axon GenePix laser scanner after washing thoroughly. The relative expression of proteins was analyzed by comparing the signal intensities. The intensities of signals were quantified by densitometry, and relative differences in expression levels of each sample were determined [8]. For quantitative fold changes, we set a fold change ≥ 2 and *p* < 0.05 as the cutoff for the differentially expressed protein selection between groups.

### 2.7. Flow Cytometry Assay

The apoptosis rate of GCs intervened by different sources of FFs both EI and TI was examined using an Annexin V-FITC/PI double-staining cell apoptosis detection kit according to the manufacturer's instruction (FAK015.50, Xinbosheng Technology Co., Ltd., Shenzhen, China). Briefly, fresh collected GCs were washed twice with PBS and immediately incubated in 10 mM DCFH-DA for 20 min at 37°C in the dark. Cells were then detected by CytoFLEX and assessed by matched CytExpert for DxFLEX software.

### 2.8. Real-Time PCR and Western Blotting Analysis

The expression of apoptosis-related proteins including apoptosis regulator Bcl-2 (BCL2), apoptosis regulator BAX (BAX), casepase-3 (CASP3), casepase-9 (CASP9), and cellular tumor antigen p53 (TP53) in GCs was analyzed by real-time PCR and Western blotting [9, 10]. PCR primer pairs for the analysis were designed and synthetized by Invitrogen Biological Technology Co., Ltd., Shanghai, China (Supplementary Table 1), and antibodies used in this research are listed in Supplementary Table 2.

### 2.9. Protein–Protein Interaction (PPI) Network Analysis

The PPI data were obtained from STRING (https://string–db.org, version 11.0) which is a database for predicting protein–protein interactions with confidence score ranges (low confidence score < 0.4, medium 0.4–0.7, high > 0.7–0.9, and highest confidence > 0.9) [11]. The target proteins were selected with species limited to “Homo sapiens” and a confidence score > 0.4 [12]. The direct or indirect interaction with specific proteins in FFs detected by protein microarray and apoptosis-related proteins in treated GCs was obtained through STRING. To investigate the functional annotation and involved pathways of these proteins, the GO- and KEGG-enrichment analyses were calculated and evaluated by DAVID version 6.8 (Database for Annotation, Visualization and Integrated Discovery, http://david.abcc.ncifcrf.gov/home.jsp) [13]. Difference was considered to be statistically significant at *p* < 0.01.

### 2.10. Statistical Analysis

Statistical evaluations were analyzed using the IBM SPSS Statistics version 23.0 and GraphPad Prism 5 software. Data were first assessed for normality and homogeneity of variance and then presented as the mean ± standard deviation (s.d.). Data were analyzed using a *t*-test or one-way, two-way, or repeated-measure ANOVA, followed by Tukey's or Dunnett's posttest. Differences were considered significant at *p* < 0.05.

## 3. Results

### 3.1. The Hormone Levels of FFs

**Figure 1:**
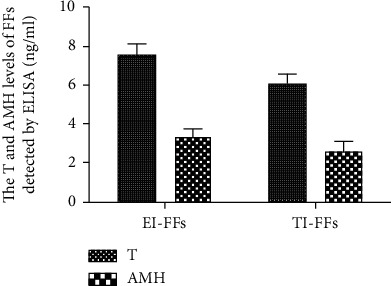
The T and AMH hormone levels of FFs in all patients were analyzed by ELISA. As is shown above, both the level of T (7.561 ± 0.582) and the level of AMH (3.361 ± 0.371) in EI-FFs were higher than those of TI-FFs (T: 6.095 ± 0.488; AMH: 2.605 ± 0.412). However, there was no obvious difference between the two groups (T: *p* = 0.585; AMH: *p* = 0.178).

The T and AMH hormone levels of 30 EI-FFs and 30 follicular fluids of tubal-associated infertility patients (TI-FFs) were tested and compared. According to the analysis of ELISA, neither the T nor AMH level was obviously different between the two groups (Figure 1).

### 3.2. Specific Proteins in FFs

**Figure 2:**
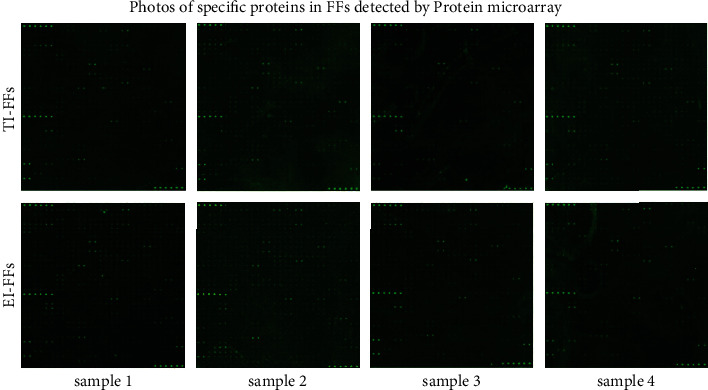
The photos of specific proteins in FFs detected by protein microarray.

**Figure 3:**
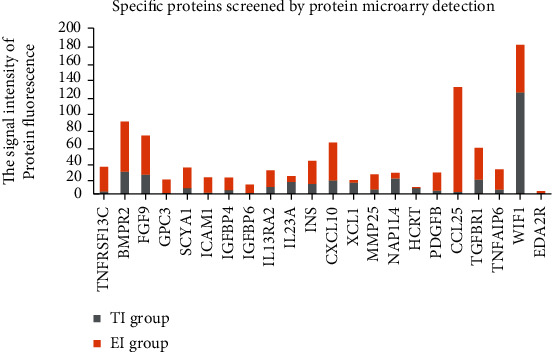
The specific proteins screened in FFs by proteomic profiling.

Eight FF samples were randomly selected from the 60 patients including 4 EI group patients and 4 TI group patients for proteomic profiling. After the fluorescence signal was interpreted by GenePix Pro 6.0 and compared with the positive reference material, the background effect was removed, and the fluorescence signal intensity of 507 proteins in each sample was obtained. With the difference of 2 times or more as the standard and *p* < 0.05 as the significant difference, a total of 22 specific proteins with significant difference were screened and named insulin (INS), glypican-3 (GPC3), interleukin-23 subunit alpha (IL23A), etc.; see Figures 2 and 3 and Supplementary Table 3 for details.

### 3.3. The Apoptosis Rate of GCs

**Figure 4:**
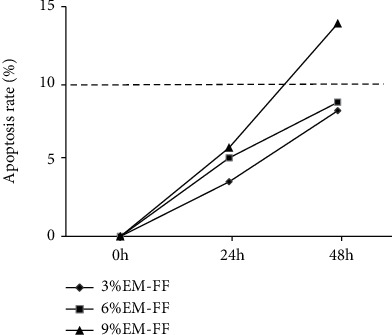
The apoptosis rates were counted after the TI-GCs were incubated in different concentrations of EI-FFs (3%, 6%, and 9%) at different times (24 h and 48 h). As is shown, 9% EI-FF intervention in TI-GCs for 48 h had an obvious effect on apoptosis according to the standard of apoptosis rate at 10% or more.

**Figure 5:**
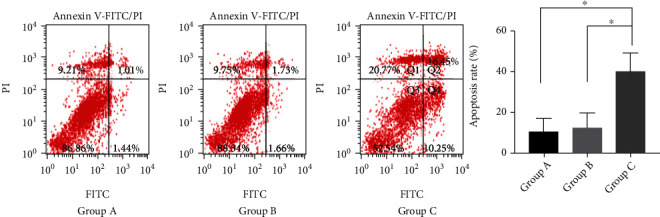
The apoptosis rates of TI-GCs analyzed by flow cytometry. Group A: TI-GCs incubated in basal medium (DMEM/F-12 supplemented with 10% fetal bovine serum); Group B: TI-GCs incubated in basal medium with 9% TI-FFs; and Group C: TI-GCs incubated in basal medium with 9% EI-FFs. After being incubated 48 h, all of the three groups were tested by flow cytometry and the apoptosis rates were compared between groups. As shown in the chart, the apoptosis rate of Group C was obviously higher than those of Group A and Group B. These suggest that 9% EI-FFs have a significant effect on TI-GC apoptosis.

The frozen FFs were taken from -80°C and thawed at room temperature. According to our design, the EI-FFs were diluted to 3%, 6%, and 9%, and the TI-FFs were diluted to 9% in the basal medium. The TI-GCs was cultured in vitro following the above method. 24 h later, the adherent growth of TI-GCs was confirmed through a microscope, and then, the cells were incubated in different concentrations of EI-FFs (3%, 6%, and 9%) and TI-FFs (9%) at different times (0 h, 24 h, and 48 h), and the apoptotic rates were analyzed by flow cytometry. According to the reference [14], the apoptosis rate at 10% or more was considered to be a standard for the cell termination, initiation, or entry into the apoptotic stage. Our study indicated that 9% EI-FF intervention in TI-GCs for 48 h had an obvious effect on apoptosis (Figure 4), and the apoptosis rate was significantly increased compared with 9% TI-FFs on TI-GCs (Figure 5).

### 3.4. The Expression of Apoptosis-Related Proteins in GCs

**Figure 6:**
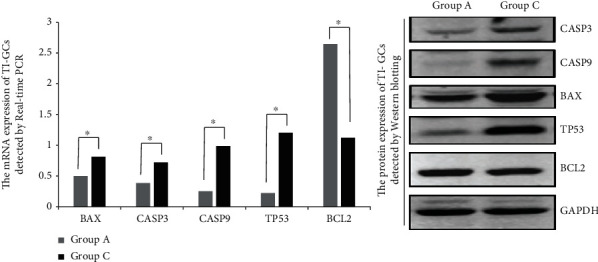
The expression of apoptosis-related proteins analyzed by real-time PCR and Western blotting. Group A: TI-GCs incubated in basal medium (DMEM/F-12 supplemented with 10% fetal bovine serum) for 48 h; Group C: TI-GCs incubated in basal medium with 9% EI-FFs for 48 h. As is shown, the expression of BAX, CASP3, CASP9, TP53 mRNA, and protein was obviously increased and the expression of BCL2 mRNA and protein was obviously decreased in Group C compared with Group A. ^∗^*p* < 0.05.

To explore the mechanisms of EI-FFs inducing the apoptosis of TI-GCs, real-time PCR and Western blotting were used to detect the expression of important apoptosis-related proteins including BCL2, BAX, CASP3, CASP9, and TP53. According to our analysis, the expression of BAX, CASP3, CASP9, and TP53 mRNA and protein was obviously increased in the group of TI-GCs incubated in 9%EI-FFs compared with the control group (TI-GCs incubated in basal medium), while the expression of BCL2 was obviously decreased. This suggests that EI-FFs can accelerate the process of TI-GC apoptosis by regulating the expression of BAX, CASP3, CASP9, TP53, and BCL2 (Figure 6).

### 3.5. The Correlation between the Specific Proteins in EI-FFs and the Apoptosis-Related Proteins in GCs

**Figure 7:**
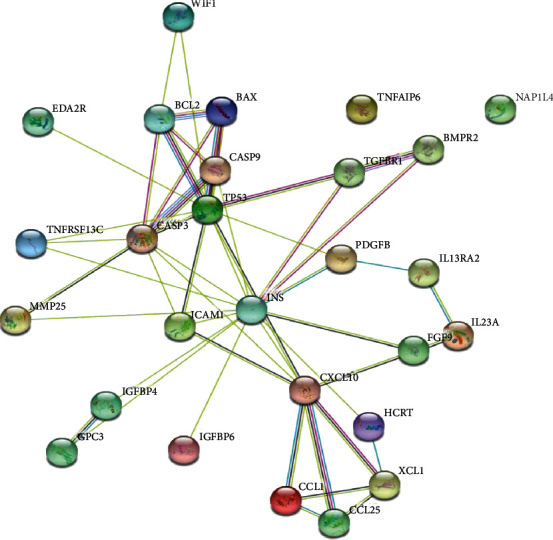
Interaction network of all proteins including 22 specific proteins in EI-FF and 5 apoptosis-related proteins in TI-GC builds by STRING online server.

**Figure 8:**
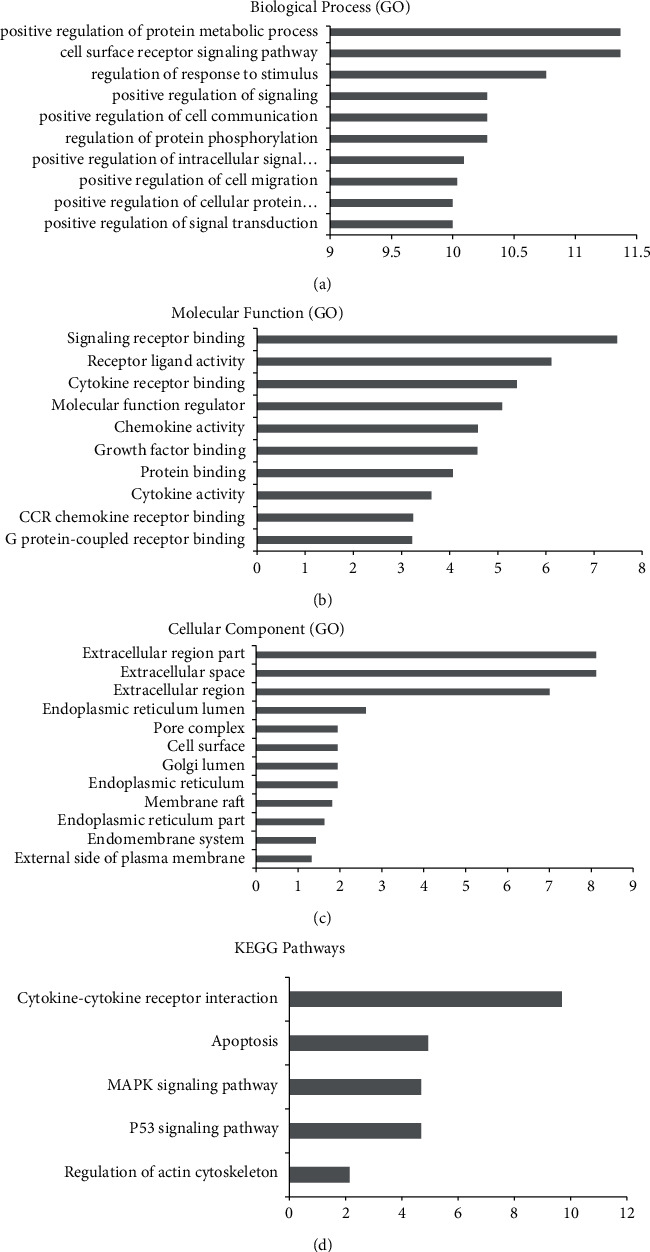
GO and KEGG analysis of targets: (a) biological process; (b) molecular function; (c) cell component; (d) KEGG pathway. The *y*-axis shows significantly enriched categories of the targets, and the *x*-axis shows the enrichment scores of these terms (*p* < 0.01).

In our research, a total of 22 proteins were specifically expressed in EI-FFs according to the proteomic profiling, and EI-FFs could accelerate the apoptosis process of TI-GCs by regulating the expression of 5 apoptosis-related proteins as described above. The STRING online server, a network database for predicting protein–protein interactions with confidence score ranges, was used to explore the correlation between 22 specific proteins and 5 apoptosis-related proteins. It was clear to discover that each specific protein, except TNFAIP6 and NAP1L4, directly or indirectly acted on the apoptosis-related proteins according to the interactive network build by STRING. Importantly, INS, CXCL10, ICAM1, WIF1, and TNFRSF13C are more closely related (Figure 7), and they were considered to be significant protein biomarkers in our study. To further investigate the multiple mechanisms of EI-FFs on TI-GCs systematically, GO-enrichment analysis for the biological process, molecular function, cellular component, and KEGG pathways of the selected proteins was performed using DAVID 6.8. The results indicated that EI-FFs induced the apoptosis of TI-GCs by regulating multiple biological processes (*p* < 0.01), and the top five of them were positive regulation of protein metabolic process (GO:0051247), cell surface receptor signaling pathway (GO:0007166), regulation of response to stimulus (GO:0048583), positive regulation of signaling (GO:0023056), and positive regulation of cell communication (GO:0010647) (Figure 8(a)). The top five molecular functions terms (*p* < 0.01) included signaling receptor binding (GO:0005102), receptor ligand activity (GO:0048018), cytokine receptor binding (GO:0005126), molecular function regulator (GO:0098772), and chemokine activity (GO:0008009) (Figure 8(b)), while the main cellular component terms (*p* < 0.01) were extracellular region part (GO:0044421), extracellular space (GO:0005615), extracellular region (GO:0005576), endoplasmic reticulum lumen (GO:0005788), and pore complex (GO:0046930) (Figure 8(c)). As shown in Figure 8(d), the selected proteins were further mapped to 5 KEGG pathways with *p* < 0.01. The data indicated that EI-FFs effected on TI-GCs primarily depending on cytokine-cytokine receptor interaction (has:04060), apoptosis (has:04210), MAPK signaling pathway (has:04010), p53 signaling pathway (has:04115), and regulation of actin cytoskeleton (has:04810), etc.

## 4. Discussion

Currently, 10%–15% of couples at reproductive age suffer from infertility, and a better understanding of the regulatory processes of reproduction could allow for the continuous improvement in the success of infertility treatment [15]. Endometriosis is an important cause of female infertility and seriously impacts the physical and psychological health of patients. Endometriosis is now considered to be a public health problem that deserves in-depth investigation [16], especially the etiopathogenesis of endometriosis-associated infertility.

Some researchers have focused on these issues and tried their best to reveal the mystery. There are pieces of evidence suggesting several different mechanisms potentially involved in endometriosis-associated infertility, including anatomical and microenvironmental conditions that may negatively impact the oocyte acquisition, egg fertilization, and zygote transport within the tube and embryo implantation. For example, the peritoneal ectopic endometrial foci can cause local inflammatory reactions, with the recruitment of macrophages, release of cytokine, and generation of reactive oxygen species, resulting in a prooxidant peritoneal microenvironment; these alterations may be systemically reflected and also affect the follicular microenvironment [17]. A harmful follicular fluid may disrupt cumulus cell functions and, consequently, compromise oocyte competence [18], and so on.

Follicular fluids (FFs) surround the granulosa cell-oocyte complex and mediate factors in the communication between the cells within the follicle [19]. Recently, different studies have investigated the changes in the FF composition of women with endometriosis, such as gonadal hormones [20], growth factors [21], and cytokines [22], and accumulating evidence suggests that the hormone microenvironment contributes to the oocyte maturation, oocyte quality, and even the subsequent embryonic development. However, there is conflicting data in the literatures concerning the level of the key FF hormones. In this case control study, neither the T nor the AMH level of FFs has obvious difference between the EI-FFs and TI-FFs. Thus, we speculated that more samples might be needed to analyze the FF hormones.

Diseases are complex systems that can be studied through the integration of data derived from different disciplines to obtain a global and reliable picture of the biological phenomenon under investigation [23]. Systems biology and biologically integrated approaches are powerful tools to analyze a disease as a whole. For this, it is necessary to collect and integrate different data types such as genes, proteins, and metabolites coming from some “omics” disciplines in order to create an information body that allows us to have a comprehensive and integrated vision of the biological phenomenon under investigation. In this context, the evaluation of the proteomic profiling is a powerful and reliable tool for the identification of total protein molecules present in different biological systems under a given physiological condition and is able to identify new diagnostic/prognostic biomarkers [24]. In our study, a total of 22 proteins with significant difference between EI-FFs and TI-FFs were screened by proteomic profiling including insulin (INS), glypican-3 (GPC3), and interleukin-23 subunit alpha (IL23A). In the EI-FF group, tumor necrosis factor receptor superfamily member 13C (TNFRSF13C), bone morphogenetic protein receptor type-2 (BMPR2), fibroblast growth factor 9 (FGF9), GPC3, C-C motif chemokine 1 (SCYA1), intercellular adhesion molecule 1 (ICAM1), insulin-like growth factor-binding protein 4 (IGFBP4), insulin-like growth factor-binding protein 6 (IGFBP6), interleukin-13 receptor subunit alpha-2 (IL-13RA2), INS, C-X-C motif chemokine 10 (CXCL10), matrix metalloproteinase-25 (MMP25), platelet-derived growth factor subunit B (PDGFB), C-C motif chemokine 25 (CCL25), TGF-beta receptor type-1 (TGFBR1), tumor necrosis factor-inducible gene 6 protein (TNFAIP6), and tumor necrosis factor receptor superfamily member 27 (EDA2R) were significantly increased, while IL23A, lymphotactin (XCL1), nucleosome assembly protein 1-like 4 (NAP1L4), orexin (HCRT), and Wnt inhibitory factor 1 (WIF1) were significantly decreased compared with the TI-FF group. According to bioinformatics analysis, we found that the molecular function of these specific proteins were mainly related to receptor ligand activity, signaling receptor binding, growth factor binding, chemokine activity, cytokine receptor binding, G protein-coupled receptor binding, and insulin-like growth factor, etc.

Moreover, GCs have been demonstrated to play an important role in determining the fate of follicles, serving molecules that are essential for follicular growth and maintenance, and killing themselves under the regulation of various cytokines, hormones, and growth factors though an apoptotic process that results in follicular dysplasia or atresia [25]. In general, proliferation and differentiation of GCs lead to follicular maturation and ovulation, whereas degeneration and apoptosis of GCs result in follicular dysfunctions and dysplasia [26]. Recently, researchers have gradually realized that GC apoptosis is the basic pathological mechanism of follicular dysplasia or atresia [3], and follicular development studies have become increasingly focused on the molecular regulation of GC apoptosis. Apoptosis, as an autonomous mode of death, plays an important role in tissue development and disease progression. Currently, studies on apoptotic mechanism of GCs mainly focus on apoptosis-related signaling pathways and apoptosis-related transcriptional/posttranscriptional regulation. It had been proven that the proliferation and apoptosis of GCs involve survival factors represented by forkhead box O (FOXO), B-cell lymphoma 2 (BCL2), and inhibitor of apoptosis proteins (IAPs), etc. and apoptosis-inducing factors such as Fas/FasL, cysteinyl aspartate specific proteinase (caspase), and TP53, which interact with each other. It is generally believed that Fas/FasL, regulated by negative feedback of gonadal hormones, plays a most important role in the apoptosis of GCs. According to reports, cell cycle arrest and high expression of TP53 could be induced if Fas expression on the GC surface is increased [27], and cell apoptosis could be promoted by caspase-8 and cytochrome C when fas-associating protein with a novel death domain (FADD) is activated [28]. In this research, we found that EI-FFs could accelerate the process of TI-GC apoptosis by upregulating the expression of BAX, CASP3, CASP9, and TP53 and downregulating the expression of BCL2. It is worth mentioning that most specific proteins directly or indirectly acted on the apoptosis-related proteins according to the interactive network build by STRING, and INS, CXCL10, ICAM1, WIF1, and TNFRSF13C are more closely related and possible for the important protein biomarkers related to this disease. Importantly, these 22 special proteins of EI-FFs and 5 apoptosis-related proteins of TI-GCs were analyzed by GO and KEGG analyses in our research, and the results indicated that EI-FFs may induce the apoptosis of TI-GCs via regulating multiple biological processes and signaling pathways, primarily relating to cytokine-cytokine receptor interaction, apoptosis, MAPK signaling pathway, p53 signaling pathway, and regulation of actin cytoskeleton. However, we can only speculate but not confirm which mechanism really has an impact, because in evidence-based medicine, the conclusion cannot be determined until precise experiments are properly conducted.

In our research, we clarified the effect of EI-FFs on inducing the apoptosis of GCs and tried our best to reveal the mechanisms of the etiopathogenesis of endometriosis-associated infertility. We established a protein network and made correlation analyses to predict the most promising protein biomarkers and signaling pathways related to this disease.

## Data Availability

The proteomic profiling data and the protein–protein interaction (PPI) network analysis data are available on the free online platform of the RayBiotech Cloud Platform (http://www.raybiotech.com) and STRING (https://www.string-db.org/cgi/network?taskId=bC6EjyxlWEaF&sessionId=bJBGCz98JXO4), respectively, with the account provided by the authors. In addition, other relevant data mentioned in our article could be found in the Supplementary Information files.

## Abbreviations

FFs: Follicular fluids
GCs: Granulosa cells
EI: Endometriosis-associated infertility
TI: Tubal-associated infertility
EI-FFs: Follicular fluids of endometriosis-associated infertility patients
TI-FFs: Follicular fluids of tubal-associated infertility patients
TI-GCs: Granulosa cells of tubal-associated infertility patients
AMH: Anti-Müllerian hormone
T: Testosterone
PPI: Protein–protein interaction
BCL2: Apoptosis regulator Bcl-2
BAX: Apoptosis regulator BAX
CASP3: Casepase-3
CASP9: Casepase-9
TP53: Cellular tumor antigen p53
INS: Insulin
CXCL10: C-X-C motif chemokine 10
ICAM1: Intercellular adhesion molecule 1
WIF1: Wnt inhibitory factor 1
TNFRSF13C: Tumor necrosis factor receptor superfamily member 13C.

## Supplementary Materials

Supplementary Materials Supplementary Table 1: the primer sequences of target genes in real-time PCR. Supplementary Table 2: the antibodies used in Western blotting. Supplementary Table 3: specific proteins in follicular fluid samples detected by proteomic profiling.
