# Supplementary material for: The Mechanism Exploration of Follicular Fluids on Granulose Cell Apoptosis in Endometriosis-Associated Infertility
Source: Biomed Res Int. 2021 Oct 28;2021:6464686. doi: 10.1155/2021/6464686 (PMC8568523; doi:10.1155/2021/6464686)
Supplement: Supplementary Materials — Supplementary Table 1: the primer sequences of target genes in real-time PCR. Supplementary Table 2: the antibodies used in Western blotting. Supplementary Table 3: specific proteins in follicular fluid samples detected by proteomic profiling. [file 6464686.f1.docx]

**Supplementary tables**

**Supplementary table 1 The primer sequences of target genes in Real-time PCR**

| Gene | F（5’-3’） | R（5’-3’） |
| --- | --- | --- |
| BCL-2 | GAGGATTGTGGCCTTCTTTG | ACAGTTCCACAAAGGCATCC |
| BAX | TTTGCTTCAGGGTTTCATCC | CAGTTGAAGTTGCCGTCAGA |
| TP53 | GTTCCGAGAGCTGAATGAGG | TCTGAGTCAGGCCCTTCTGT |
| CASP9 | CTAGTTTGCCCACACCCAGT | GCATTAGCGACCCTAAGCAG |
| CASP3 | TGGAATTGATGCGTGATGTT | GGCAGGCCTGAATAATGAAA |
| β-actin | ACCCACACTGTGCCCATCTATG | AGAGTACTTGCGCTCAGGAGGA |

The expression of apoptosis-related proteins including BCL2, BAX, CASP3, CASP9, TP53 in GCs were analyzed by Real-time PCR. PCR primer pairs for the analysis were designed and synthetized by Invitrogen Biological Technology Co., Ltd., Shanghai.

**Supplementary table 2 The antibodies used in Western Blotting**

| Antibody | Host species | Source | No. |
| --- | --- | --- | --- |
| BCL-2 | Rabbit | Affinity Biologicals | AF6139 |
| BAX | Rabbit | Affinity Biologicals | AF0120 |
| TP53 | Rabbit | Affinity Biologicals | AF0879 |
| CASP9 | Rabbit | Affinity Biologicals | AF6348 |
| CASP3 | Rabbit | Affinity Biologicals | AF6311 |
| GAPDH | Rabbit | Affinity Biologicals | AF7021 |

Specific proteins in Follicular fluids were analyzed by proteomics profiling, according to a fold change ≥2 and *P*<0.05, a total of 22 specific proteins with significant difference were screened between the endometriosis-associated infertility group patients and the tubal-associated infertility group patients, and [respectively](javascript:;)  named Insulin (INS), Glypican-3 (GPC3), Interleukin-23 subunit alpha (IL23A), etc., see Supplementary Table 3 for details.

**Supplementary table 3 Specific proteins in Follicular fluids samples detected by proteomics profiling**

| **Protein names** | **gene name** | **TI group** | **EI group** | **EI group /TI group** | ***P* value** |
| --- | --- | --- | --- | --- | --- |
| Tumor necrosis factor receptor superfamily member 13C | TNFRSF13C | 3.313744 | 29.23979 | 8.823792665 | 0.01 |
| Bone morphogenetic protein receptor type-2 | BMPR2 | 26.995 | 60.25327 | 2.232015929 | 0.01 |
| Fibroblast growth factor 9 | FGF9 | 23.29814 | 47.13206 | 2.022996686 | 0.04 |
| Glypican-3 | GPC3 | 1.008531 | 16.34194 | 16.20370618 | 0.00 |
| C-C motif chemokine 1 | SCYA1 | 7.124879 | 24.58266 | 3.450256489 | 0.00 |
| Intercellular adhesion molecule 1 | ICAM1 | 1.604484 | 18.31534 | 11.41509669 | 0.03 |
| Insulin-like growth factor-binding protein 4 | IGFBP4 | 4.583065 | 15.15425 | 3.306575403 | 0.03 |
| Insulin-like growth factor-binding protein 6 | IGFBP6 | 0.399199 | 10.70525 | 26.81682569 | 0.01 |
| Insulin-like growth factor-binding protein 6 | IL13RA2 | 8.375369 | 20.2421 | 2.416860678 | 0.02 |
| Interleukin-23 subunit alpha | IL23A | 14.4372 | 7.060419 | 0.489043513 | 0.02 |
| Insulin | INS | 12.2541 | 27.44408 | 2.239583486 | 0.02 |
| C-X-C motif chemokine 10 | CXCL10 | 16.43096 | 45.5545 | 2.772479514 | 0.00 |
| Lymphotactin | XCL1 | 13.61662 | 2.971373 | 0.218216635 | 0.02 |
| Matrix metalloproteinase-25 | MMP25 | 5.562404 | 18.15617 | 3.264086895 | 0.04 |
| Nucleosome assembly protein 1-like 4 | NAP1L4 | 18.72305 | 6.766282 | 0.361387808 | 0.03 |
| Orexin | HCRT | 6.94957 | 1.24599 | 0.179290229 | 0.04 |
| Platelet-derived growth factor subunit B, PDGF subunit B | PDGFB | 4.125058 | 21.50035 | 5.212132775 | 0.03 |
| C-C motif chemokine 25 | CCL25 | 2.528261 | 126.4443 | 50.01236027 | 0.05 |
| TGF-beta receptor type-1 | TGFBR1 | 17.37496 | 38.33671 | 2.206434432 | 0.03 |
| Tumor necrosis factor-inducible gene 6 protein | TNFAIP6 | 5.057168 | 24.65433 | 4.875125762 | 0.04 |
| Wnt inhibitory factor 1 | WIF1 | 122.5353 | 57.55726 | 0.469719828 | 0.03 |
| Tumor necrosis factor receptor superfamily member 27 | EDA2R | 0 | 3.539906 | 0! | 0.00 |
